# Metagenomic 16S rRNA Sequencing Analysis of Pacific Oyster (*Crassostrea gigas*) Microbiota from the Puget Sound Region in the United States

**DOI:** 10.1128/genomeA.00468-17

**Published:** 2017-06-08

**Authors:** Zhen Li, Leyi Wang

**Affiliations:** a Washington State Department of Health, Public Health Laboratories, Shoreline, Washington, USA; b Ohio Department of Agriculture, Animal Disease Diagnostic Laboratory, Reynoldsburg, Ohio, USA

## Abstract

This is the first study analyzing Pacific oyster microbiota in the Puget Sound estuarine system using a next-generation sequencing method. Taxonomic analysis indicated that *Tenericutes, Chlamydiae*, *Proteobacteria*, and *Firmicutes* were the most abundant phyla. Small numbers of operational taxonomic units (OTUs) belonging to the *Vibrio* genus were detected in all the oyster microbiome samples.

## GENOME ANNOUNCEMENT

Harvesting of Pacific oysters (*Crassostrea gigas*) is an important component of the maritime economy in the Puget Sound region. Because of their filter-feeding behavior, oysters contain complex microbial populations, which could contribute to oyster summer mortalities as well as food-related outbreaks. In this study, we analyzed *C. gigas* microbiota in the Puget Sound estuarine system of the U.S. Pacific coast. Oyster samples were collected during low tide at Hood Canal (47.66585, -122.90285) and Oakland Bay (47.228614, -123.063575) growing areas in the Puget Sound estuarine system from July to October 2014. A total of 13 Hood Canal samples and 11 Oakland Bay samples were collected over this period of time. Oysters were shipped on ice gel to the lab and processed within 24 h of collection. The oysters were washed and shucked according to the FDA Bacteriological analytical manual procedure (1). To make one pooled sample, 10 to 12 adult oysters were shucked. Oyster tissue and hemolymph were transferred to a sterile container and then homogenated with an equal weight of sterile phosphate-buffered saline in a blender for 2 min. DNA was extracted using the Qiagen DNeasy blood and tissue kit (Qiagen, Valencia, CA).

The paired-end sequencing was performed using an Illumina MiSeq sequencer with the Illumina MiSeq reagent kit 600 version 3, according to the Illumina 16S metagenomic library preparation protocol (2) and the 16S rRNA primer set targeting the V3 and V4 regions of the 16S rRNA gene (3). The numbers of raw paired-end reads ranged from 79,174 to 2,497,062 in these oyster samples. Sequences were analyzed via a non-operational taxonomic unit (non-OTU) binning method. Raw paired-end reads were quality trimmed, normalized to 70,000 paired-end reads per sample using the FASTQ Toolkit version 2.0.0 on the BaseSpace Sequence Hub, and assembled using PANDAseq (4). Chimeric sequences were detected using the Decipher chimera detection tool (5) and removed. Taxonomy was assigned to each sequence using the Classifier program in Ribosomal Database Project (RDP) 11.1 (6). Taxonomic abundance analysis was also performed using the Metagenomics RAST (MG-RAST) server (7), and the best-hit classification was used via the RDP database, with the maximum E value cutoff at 1e^-10^, minimum identity cutoff at 60%, and minimum alignment length cutoff at 15. *Tenericutes* (9.79 to 56.58%), *Chlamydiae* (0.71 to 47.18%), *Proteobacteria* (2.21 to 23.29%), and *Firmicutes* (1.10 to 72.70%) were identified as the most abundant phyla in the oyster microbiota from both Hood Canal and Oakland Bay sites. OTUs that belong to the *Vibrio* genus were detected from all samples, ranging from 13 to 951 OTU per oyster sample, indicating the persistence of extremely small population of *Vibrio* spp. in healthy oyster microbiota. Further analysis will be focused on the variations in oyster microbiome samples collected at different times, temperatures, and locations.

### Accession number(s).

Raw sequences were deposited to the NCBI SRA database under accession no. SRP075291. Filtered and assembled sequences are also publicly available on the MG-RAST server under the project ID mgp15218 and MG-RAST ID mgm4666675.3 to mgm4666698.3.
